# A Case of Hæmorrhage Following the Extraction of a Tooth

**Published:** 1869-12

**Authors:** J. D. Patterson

**Affiliations:** Lawrence, Kansas.


### ARTICLE II.

### *A Case of Hæmorrhage Following the Extraction of a Tooth.*

By Dr. J. D. Patterson, Lawrence, Kansas.

I have lately had a case of severe hæmorrhage from the socket of an extracted tooth, which I deem might be of interest to you and the *Journal.* The patient, Rev. M. A., of this city, called at my office on Friday to have the first inferior molar of the right side extracted. He has been suffering from impaired health and lacks *tone* in the system, The tooth being badly diseased I extracted it. The bleeding was profuse but I supposed it nothing more than usual as the parts were turgid with blood, and sent the patient away. On the following Saturday he called at 9 A. M. Bleeding from the socket had continued incessantly, but slight. I removed the clot and filled the cavity with cotton and tannin, and the bleeding was stopped. Monday morning the patient called with very severe hæmorrhage, and stated that during the night the bleeding returned, saturating the parts of his bed near the head thus awaking him. When he came to the office he was unable to walk without staggering being so feeble from loss of blood, his sight also was dimmed. I cleaned the cavity, removing the old pellets and plugged with cotton charged with "*Monsel's sulphas ferri*", used pressure with the thumb until bleeding was stopped, dried the parts and coated with plaster of paris, keeping dry till hard and discharged the patient. No return of bleeding. The plaster for thirty hours kept hard and seemed to do as well as any compress could, besides, having the advantage of being easily and quickly applied.

